# A Prospective, Non-interventional Observational Study to Assess the Efficacy, Safety, and Tolerability of the Casein Hydrolysate-Based Food Supplement in High-Risk Human Papillomavirus-Positive Women

**DOI:** 10.7759/cureus.80201

**Published:** 2025-03-07

**Authors:** Carmen Pingarron, Alfonso Duque, Ana López, Juan Ferragud

**Affiliations:** 1 Obstetrics and Gynecology, Hospital Quirón Salud San José, Madrid, ESP; 2 Obstetrics and Gynecology, Hospital Ruber Internacional, Madrid, ESP; 3 Obstetrics and Gynecology, Hospital HM Vallés, Madrid, ESP; 4 Obstetrics and Gynecology, NTD Labs, Terrassa, ESP

**Keywords:** casein hydrolysate, cervical lesions, clearance, ditriamino, hpv, human papillomavirus, hupavir, immunomodulation

## Abstract

Objective

The study aimed to assess the efficacy, safety, and tolerability of casein hydrolysate-based food supplement, HuPaVir^®^, in high-risk human papillomavirus (HR-HPV)-positive women.

Methods

A prospective, multicenter, and non-interventional observational study was carried out in Spain. At the end of the study, 118 HR-HPV-positive women (20 to 65 years old) were enrolled and completed the follow-up as required by the protocol. All participants performed HPV-DNA testing, liquid-based cytology, and DNA extraction by multiplex PCR at zero and six months. Upon baseline evaluation, participants were provided with a casein hydrolyzed formula or managed with a wait-and-see approach. Supplementation treatment consisted of daily consumption of one dose of the casein hydrolysate formula for six months. The study population was then divided into four groups based on the baseline cytology result and the supplementation treatment performed during the follow-up period. Treated participants underwent blood tests to determine different immune parameters. Treated participants were also provided with case report forms to register any adverse effect during the study and were asked to complete a voluntary survey to assess product tolerance and acceptability by rating their level of agreement.

Results

At six months, consumption of the casein hydrolysate formula resulted in total or partial clearance of the HR-HPV type and normalization of cervical cytology in a statistically significant percentage of cases compared to the untreated group (74.6% vs. 35.6%; p<0.01 and 67.4% vs. 41.9%; p<0.05, respectively). Notably, a positive trend in increasing serum levels of innate immunity markers, such as natural killer (NK) cells, and adaptive immunity markers, such as CD8 and CD4 T lymphocytes, was observed in the treated participants.

Conclusion

The results suggest that daily supplementation with the casein hydrolysate formula is effective in causing significant virus clearance and lesion remission compared to the conventional approach in clinical practice for HR-HPV-positive women. However, a large, multicenter, standardized, and randomized study with longer-term follow-up, 12 months after supplementation treatment, is needed to confirm and strengthen these findings. The formula was found to be safe and well-tolerated.

## Introduction

Human papillomavirus (HPV) is a double-stranded DNA virus that lacks a lipid envelope. There are over 100 types of HPV, which are classified as either cutaneous or mucosal based on the tissue they infect, and as low-risk (LR-HPV) or high-risk (HR-HPV) based on their association with cancer development and their infection persistence [1,2]. In this sense, 15 HPV types are considered high-risk types (16, 18, 31, 33, 35, 39, 45, 51, 52, 56, 58, 59, 68, 73, and 82) while three are classified as probable high-risk types (26, 53, and 66) [3].

HPV infection is acquired through direct contact with the skin or mucous membranes of an infected person, even in the absence of visible lesions. The primary mode of transmission for genital infection is sexual contact [4]. HPV infection is very common, and it is estimated that approximately 80% of women in the United States will have acquired it by the age of 50 [5].

Most HPV infections do not cause symptoms or disease and resolve spontaneously between 12 and 24 months after infection. However, a small proportion of these infections can persist and lead to precancerous lesions that may eventually progress to cancer [6]. HPV infection is associated with virtually 100% of cases of cervical cancer and with a high rate of cases of anogenital and oropharyngeal cancer [7].

According to the World Health Organization (WHO), the approach to cervical cancer prevention is based on HPV vaccination (primary prevention) to avoid HPV infection and screening programs (secondary prevention) for the early detection of HPV infection [8]. Screening programs vary by country and typically involve HPV-DNA detection tests to identify the presence of the virus and cytology (Pap test) to assess for intraepithelial cellular lesions. The positive HPV-DNA test implies the presence of the virus in the sample, while the positive cytology implies an alteration or injury in the tissue [9].

The morphology of squamous intraepithelial lesions caused by HPV in the lower anogenital tract is identical in all locations and in both sexes [10]. The Lower Anogenital Squamous Terminology (LAST) classifies the histological intraepithelial squamous lesions associated with HPV in two grades, low‐grade squamous intraepithelial lesions (LSIL) and high-grade squamous intraepithelial lesions (HSIL) [11]. The term LSIL also includes cervical intraepithelial neoplasia of grade 1 (CIN1) of the Richart classification, adopted by the WHO in 2004 [12].

LSIL/CIN1 lesions are the histological manifestation of a self-limited infection by HPV, which most times resolves spontaneously [10]. The strict follow-up of patients with LSIL lesions minimizes the risk of developing cervical cancer since it allows to observe if the lesions become resolved or, on the contrary, to detect early if they progress to HSIL. The lesions of CIN2 and CIN3 are included in the term HSIL [13]. The lesions HSIL/CIN2 can still return to LSIL or progress to neoplasia. In contrast, HSIL/CIN3 lesions are considered to be true intraepithelial neoplasms with a high potential for progression. They constitute the necessary precursor lesion of cervical cancer and should be treated by destructive or excisional methods [8].

Atypical squamous cell of undetermined significance (ASCUS) is another cytological alteration that may be caused by HPV infection or other factors. When detected, an HPV-DNA test is recommended. In general, the presence of ASCUS is related to SIL lesions, primarily LSIL, though HSIL cannot be ruled out [14].

The immune system is the body’s natural defense mechanism against HPV and other pathogens. It prevents the persistence and the progression of infections caused by HPV. Previous studies report suggest that while the presence of an oncogenic HPV is a risk factor, additional factors are needed for malignant progression [13].

The adequate nutritional status of patients with HPV infections is essential for the optimal functioning of the immune system. For this reason, within the strategies of observational treatment of patients with HPV infections, it is recommended to maintain an adequate diet, stop smoking, and practice regular exercise [15]. In some cases, supplementation with relevant micro and macronutrients may be beneficial to stimulate the immune system and accelerate HPV clearance and lesion resolution. Deficiencies of nutrients such as folates, vitamin C, vitamin B12, zinc, and others have been linked to increased persistence of HPV infections and progression of HPV-related lesions. Amino acids and fatty acids also contribute significantly by supporting protein synthesis and can help maintain optimal immune function and reduce the risk of infections. Both depletion of micro- and macronutrients could impair immune function by having detrimental effects on several components of the innate and adaptive immune response mechanisms involved in the defense against and clearance of HPV infection. The failure of this mechanism over a long period may further contribute to the persistence and extensive manifestations of HPV infection.

The main objective of this study is to evaluate the benefits of the supplementation with a specific casein hydrolysate-based formula to favor the clearance of HR-HPV infections and their derived lesions. This formula contains a mixture of dipeptides and tripeptides that are source of fast systemic absorption amino acids and bioactive peptides, along with vitamins (vitamins A, B3, and B9) and minerals (zinc). Deficiencies in these nutrients have been related to greater persistence of HPV infections and progression of the derived lesions [16-21].

This article was previously published on the Pre-prints.org server on 3 December 2019-https://www.preprints.org/manuscript/201912.0029/v1 and was previously presented as an abstract at the 2020 AEPCC Annual Scientific Meeting on 17, 19 and 24, 26 November 2020 Asociación Española de Patología Cervical y Colposcopia - https://www.aepcc.org/congreso2020/posters-e/

## Materials and methods

Study design and site

This was a prospective, multicenter, controlled observational study involving high-risk human papillomavirus (HR-HPV)-positive women with or without HPV-related low-grade cytological alterations who attended the gynecologist's office between July 2018 and July 2019. Three Spanish hospitals participated in the study.

One hundred and eighteen participants were considered for observation in this study. The product under study is a food supplement marketed under the name of HuPaVir®, which contains 4.5 g of patented casein hydrolysate (Ditriamino®), 120 µg (15% RDI) vitamin A, 30 µg (15% RDI) vitamin B9 (folic acid), 2.4 mg (15% RDI) vitamin B3 (niacin), and 1.5 mg (15% RDI) zinc for each dose of 6 g (NTD Labs, Terrassa, Spain).

Inclusion of participants

The study was carried out in accordance with the principles of the recommendations included in the guideline for cervical cancer prevention of the Spanish Association of Cervical Pathology and Colposcopy (AEPPC or Asociación Española de Patología Cervical y Colposcopía) without any modification of the usual clinical practice [10]. All participants gave their informed consent for inclusion before they voluntarily participated in the study in accordance with the Declaration of Helsinki. This observational study meets all legal requirements stipulated in the country where it was carried out, Spain.

The inclusion criteria were as follows: participants with a positive DNA tests for at least one HR-HPV (HPV 16, 18, 26, 31, 33, 35, 39, 45, 51, 52, 53, 56, 58, 59, 66, 68, 73, and 82) with normal ASCUS or LSIL cytology. Women from 20 to 65 years old. The exclusion criteria included participants who did not present a positive test for at least one HR-HPV.

Supplementation treatment groups and procedures

At the beginning of the study, the study population was divided into four groups as detailed in Table 1, depending on the cytology result at baseline (time 0 months) and the treatment performed during the follow-up period.

**Table 1:** Study population groups and characterization HR(+)/Cyto(-): HR-HPV-positive women without cytological alterations; HR(+)/Cyto(+): HR-HPV-positive women with HPV-related low-grade cytological alterations. Treatment: Subjects with supplementation treatment included consuming HuPaVir® (1 dose/day) every day for six months from the start of the study. No treatment (Control): Subjects received observational follow-up for six months without altering the conventional approach in clinical practice and without supplementation treatment. HPV: human papillomavirus; ASCUS: atypical squamous cells of undetermined significance; LSIL: low‐grade squamous intraepithelial lesions

Study population groups	Groups characterization	Subjects (n)	Average age
Treatment: HR(+)/Cyto(-)	Positive subjects for the HR-HPV DNA test but with normal cytology. Supplementation treated subjects. Follow-up after 6 months	30	38.5
No treatment (Control): HR(+)/Cyto(-)	Positive subjects for the HR-HPV DNA test but with normal cytology. Untreated subjects, as the control. Follow-up after 6 months	14	35.4
Treatment: HR(+)/Cyto(+)	Positive subjects for the HR-HPV DNA test but with altered cytology (ASCUS/LSIL). Supplementation treated subjects. Follow-up after 6 months	43	37.5
No treatment (Control): HR(+)/Cyto(+)	Positive subjects for the HR-HPV DNA test but with altered cytology (ASCUS/LSIL). Untreated subjects, as the control. Follow-up after 6 months	31	33.9

The diagnostic procedure was performed by liquid cytology and DNA extraction for identification of viral DNA from part of the L1 region of HPV by multiplex PCR (Linear Array HPV Genotyping Kit-Roche Diagnostics Ltd., UK). All participants included in the study were followed up after the initial visit, attending medical visits at zero and at six months, during which they underwent cervical cytology and a DNA tests for HR-HPV.

Physicians provided information about the food supplement HuPaVir® to their patients. Participation in the treatment arm was voluntary for those interested in the supplement. Patients who declined the supplement but consented to participate were included in the control group. Participants with supplementation treatment included in this study received the recommendation of consuming HuPaVir® (1 dose/day) separated from meals every day for six months from the start of the study. Furthermore, they could not jointly use other topical or ingested alternative therapies aimed at fighting HPV infection that are not included in the AEPCC clinical guide. Cervical cytology and DNA tests for HR-HPV were performed at times zero and six months. Control participants received observational follow-up without altering the conventional approach in clinical practice and without supplementation treatment. As supplemented participants, control participants underwent cervical cytology and DNA tests for HR-HPV at times zero and six months.

For participants in the supplementation treatment groups who underwent blood tests during visits at month zero or six, the determination of various immune parameters was requested. These immune parameters include levels of natural killer (NK) cells, TCD4+, TCD8+, total lymphocytes, cortisol, IGF1, and IGM. A survey was conducted and voluntarily completed by participants consuming HuPaVir® to investigate tolerance to the product and other improvements in general conditions.

Study endpoints and parameters

The primary endpoint of this study included the clearance degree of HR-HPV infections and their derived cytological alternations after six months of supplementation treatment. HR-HPV clearance was defined as a negative HPV test for the individual HPV type following a positive test for that type [22]. Total HR-HPV clearance was defined as a negative HPV test or disappearance of all types detected at baseline, whereas partial HR-HPV clearance was defined as a negative HPV test or disappearance of at least one type detected at baseline. Total or partial HR-HPV clearance was determined by the sum of the total and partial clearances. For cytological alternations, the reading of liquid‐based cytology testing was classified as normal, atypical squamous cells of undetermined significance (ASCUS), and LSIL or HSIL, following the Bethesda classification system for reporting cervical cytologic diagnoses [11].

The secondary endpoint of this study included the assessment of the nutritional supplementation effect on mediators of cellular immunity in HR-HPV-positive participants. Serum immune parameters such as NK cells, cytotoxic T lymphocytes (CD4 and CD8), immunoglobulins (IgG/IgM), insulin-like growth factor 1(IGF1), and Cortisol levels were analyzed. The participants’ degree of satisfaction with the efficacy and tolerability of the supplementation treatment HuPaVir® was assessed by filling out a voluntary survey. Complete data collection of HPV clearance, cytological alternations, and immune parameters was performed at baseline and after six months (except the participants’ degree of satisfaction was performed at baseline and after three and six months). 

Statistical analysis

The statistical analysis was performed using the statistical software Prism GraphPad (version 5.0a; Dotmatics, Boston, US). For efficacy analysis, comparisons between supplemented and control groups were conducted using the Chi-square test with a two-sided analysis and a confidence interval of 95%. Because this was the primary objective of the study, data from the supplementation treatment groups A and C and untreated control groups C and D were also pooled into two single total groups for the main analysis. For the analysis of the immune parameters, the Student t-test was conducted with a two-sided analysis and a confidence interval of 95%. The statistical significance was set at a value of p<0.1 or less.

Ethics

This is a non-interventional, observational, prospective study with a commercial food/dietary supplement product, where approval of protocol from the local Institutional Review Board (IRB) or another appropriate ethical committee has not been needed or required. The study protocol and data collection does not fit within any of the conditions which are defined in the Spanish Royal Decree 957/2020, of 3 November, regulating observational studies with medicinal products for human use, as the participants in this study will receive the same treatment, diagnostic, and monitoring procedures as they would have received if they were not included in this study. In Spain, food/dietary supplements are regulated by Royal Decree 1487/2009, which transposed Directive 2002/46/EC on the approximation of the laws of the Member States relating to food supplements into Spanish law.

## Results

At the end of the study, 118 were found to meet the inclusion criteria and completed the follow-up as required by the protocol. The study population groups and characterization concerning the supplementation treatment performed during the follow-up period are reported in Table 1. All participants were positive for at least one type of HR-HPV. Of the participants, 46.7% (55/118) presented with simultaneous infection by more than one HR-HPV type. In the supplementation treatment groups (A and C) and the control groups (B and D), infections with multiple HR-HPV types were present in 50.7% (37/73) and 40% (18/45) of the participants, respectively.

High-risk human papillomavirus

**Figure 1:**
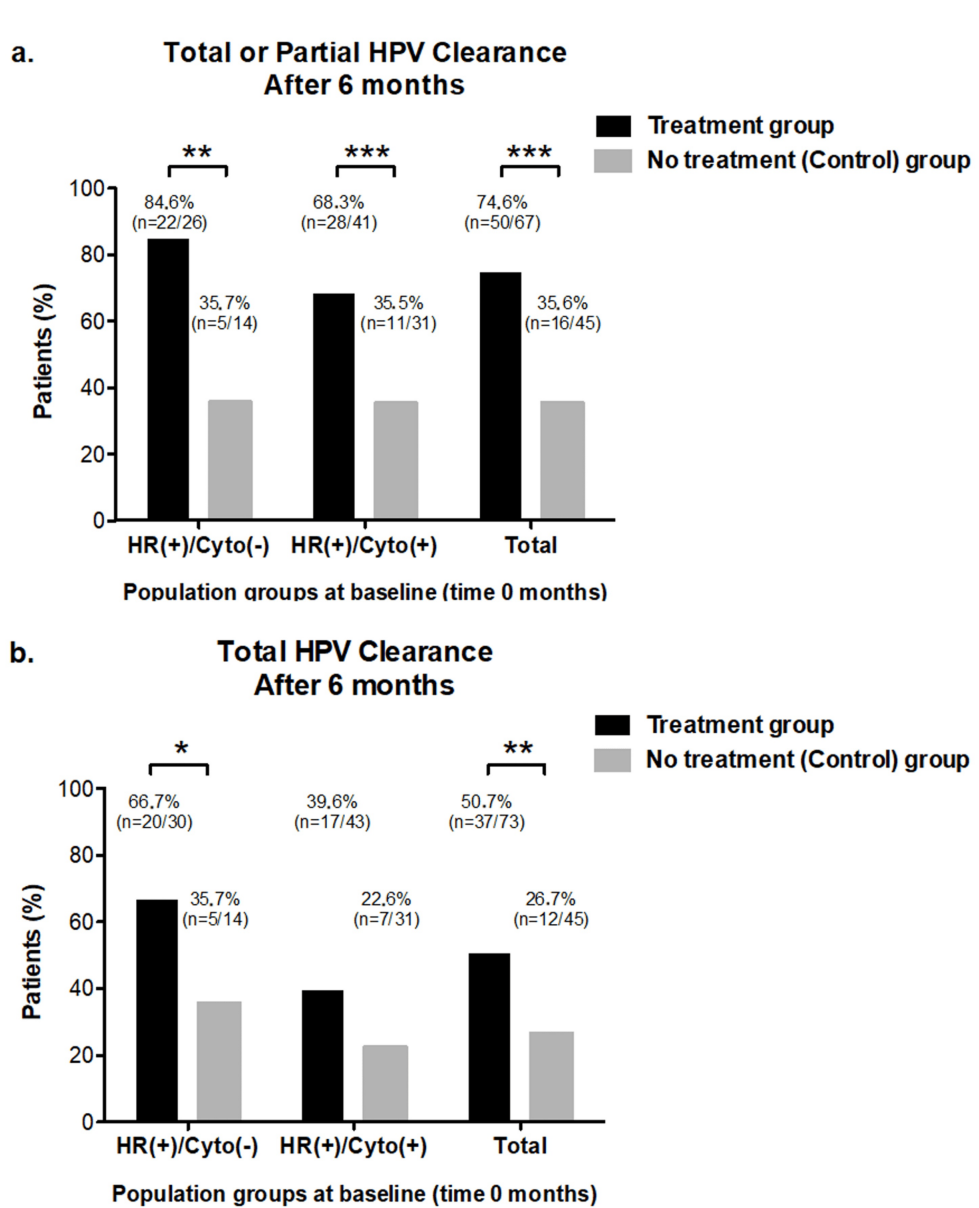
HR-HPV clearance rate ^*^p<0.1. ^**^p < 0.05. ^***^p < 0.01. Total or partial HR-HPV clearance rate (a) and total HR-HPV clearance rate (b) after six months in the HR(+)/Cyto(-) and HR(+)/Cyto(+) study population groups or in the total pooled population groups. Colored bar chart: supplementation treatment (black) and control (grey) groups at six months. Values are expressed as a percentage (%) of the number of participants at baseline (time zero months). HR-HPV: high-risk human papillomavirus

In this study, we evaluated both total and total or partial clearance of any HR-HPV at six months of follow-up (Figure 1).

Of the 118 participants, the DNA test results of six indicated the presence of one or more HR-HPV types but did not specify which types, so these participants could not be included in the analysis of partial HR-HPV clearance. For the remaining participants, total or partial HR-HPV clearance at six months of follow-up was observed in 84.6% of cases (22/26) in the supplementation treatment (A) group, compared to 35.7% (5/14) in the control (B) group among those HR-HPV-positive participants with normal cytology (HR(+)/Cyto(-)). In participants with altered cytology (HR(+)/Cyto(+)), HPV clearance was observed in 68.3% of cases (28/41) in the supplementation treatment (C) group, compared to 35.5% (11/31) in the control (D) group. When this data was pooled into a single supplementation treatment group and control group for analysis, 74.6% (50/67) vs. 35.6% (16/45) of total or partial HR-HPV clearance was observed, respectively. Overall, at six months, the percentage of participants with HPV clearance was significantly higher among the treated participants compared to those who were untreated (Figure 1a).

With respect to total HR-HPV clearance, complete clearance of all HR-HPV types at six months of follow-up was observed in 66.7% (20/30) of the supplementation treatment (A) group vs. 35.7% (5/14) of the control (B) group of those HR-HPV positive participants who were with normal cytology (HR(+)/Cyto(-)), with a noticeable tendency toward significant differences between both groups (p<0.1). As for those participants who were with altered cytology (HR(+)/Cyto(+)), a tendency toward statistical differences was observed between the supplementation treatment (C) group in comparison to the control (D) group (39.6% (17/43) vs. 22.6% (7/31), respectively). When considering the total number of treated participants compared to untreated ones, 50.7% (37/73) vs. 26.7% (12/45) of total HR-HPV clearance was observed, with this difference being statistically significant (Figure 1b).

Intracervical lesions evolution

**Figure 2:**
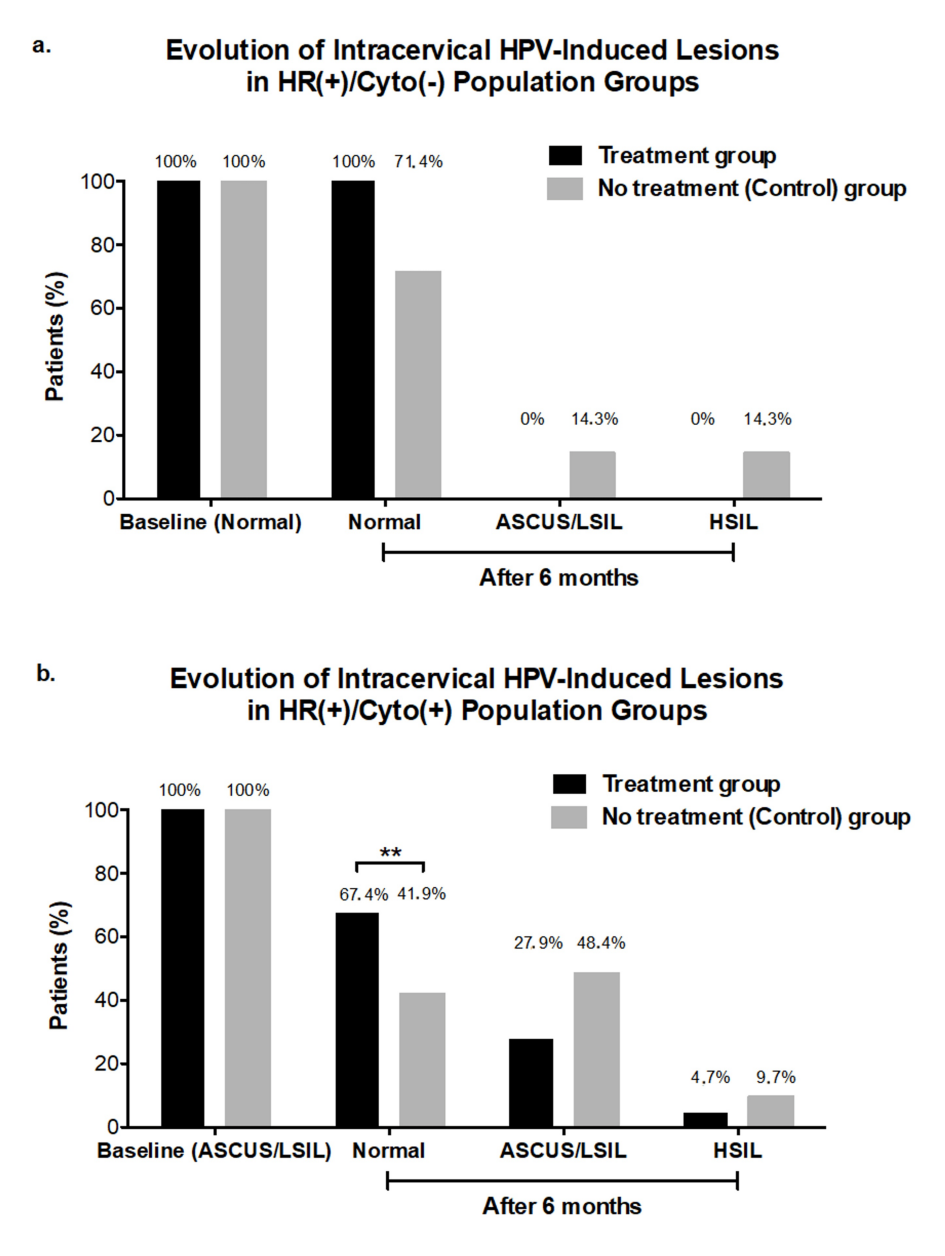
Evolution of intracervical HPV-induced lesions ^*^p < 0.1.^ **^p < 0.05. ^***^p < 0.01. Intracervical lesions evolution after six months of those participants populations who were with normal cytology (HR(+)/Cyto(-)) (a) or with altered cytology (HR(+)/Cyto(+)) (b) in the supplementation treatment and the control groups at baseline (time zero month). Colored bar chart: supplementation treatment (black) and control (grey) groups. Values are expressed as percentage (%) of the number of participants in each group. HPV: human papillomavirus; ASCUS: atypical squamous cells of undetermined significance; LSIL: low‐grade squamous intraepithelial lesions

In this study, the appearance of cervical lesions was evaluated after six months of follow-up in participants who had normal cytology at the start of the study. On the other hand, participants who had ASCUS or LSIL lesions at month zero were evaluated after six months in order to observe if lesions persisted, progressed, or underwent remission (Figure 2).

In the HR-HPV with normal cytology participants groups, a non-statistically significant trend toward a higher participants percentage was observed to remain with normal cytology at six months in the supplementation treatment group than in the control group (100% (30/30) vs. 71.4% (10/14) see Figure 2a), being noticeable a disease progression, to low (ASCUS/LSIL) or even high-grade (HSIL) lesions, was observed in the participants of the control group (14.3% (2/14) and 14.3% (2/14), respectively; see Figure 2a).

When it comes to the HR-HPV participants with altered cytology, a statistically significant difference was observed between the percentage of participants with regression toward normalized cytology at six months in the supplementation treatment group compared to the control group (67.4% (29/43) vs. 41.9% (13/31); p<0.01; Figure 2b). Although the differences were not statistically significant, a trend toward a lower percentage of participants was also observed at six months in the supplementation treatment group compared to the control group for those with altered cytology (ASCUS/LSIL lesion) (27.9% (12/43) vs. 48.4% (15/31), respectively; Figure 2b). A progression toward HSIL lesions was observed in only 4.7% (2/43) and 9.7% (3/31) of participants with altered cytology in the supplementation treatment and control groups, respectively (Figure 2b).

Effect of nutritional supplementation on the systemic immune system

To evaluate the effect of nutritional supplementation on the systemic immune system, we analyzed the blood tests available for participants supplemented with casein hydrolysate-based formula. We had a total of 12 analytics that coincided with the start of the study and another 10 analytics that coincided with the end of it. The immune parameters mean levels at months zero and six and the increase during that period is represented in Table 2 for NK cells; TCD4+ and TCD8+ lymphocytes as percentage of whole white blood cells; total lymphocytes count as well as cortisol; IGF1; IgM and IgG concentration in serum level. Differences between month zero and month six were found to be non-significant in all cases.

**Table 2:** Whole blood and serum immune parameters increase after six-month treatment NK: natural killer; IgG: immunoglobulin G; IgM: immunoglobulin M; IGF1: insulin-like growth factor 1

Parameter	Month 0	Month 6	Increase (%)
NK cells (%)	9.1	10.5	+15.7
TCD4+ lymphocytes (%)	47.2	55.6	+17.6
TCD8+ lymphocytes (%)	26.4	31.1	+17.6
Total lymphocytes (cells/µL)	2308	2497	+8.2
Cortisol (µg/dL)	14.9	18.8	+26.20
IGF1 (ng/dL)	149.2	165.0	+10.6
IgM (mg/dL)	136.8	148.9	+8.8
IgG (mg/dL)	1149.3	980.9	-14.7

Tolerability and other effects on the subject’s general condition

A survey was conducted and voluntarily answered by participants who received casein hydrolysate-based formula supplementation to investigate tolerance to the product and other effects on their general condition. Information on tolerability was available for a total of 20 participants who reported whether they had noticed any adverse effects during the six months of supplementation. Of these, 95% (19/20) reported no adverse effects, while 5% (1/20) indicated noticing a sensation of abdominal swelling, though it did not prevent them from continuing with the supplementation. Eighteen participants answered the question of whether they had observed any other improvements in their general condition. Of these, 50% (9/18) reported other improvements, including a lower incidence of colds in 22.2% of cases (4/18), greater vitality in 22.2% (4/18), and fewer recurrences of cold sores in 11.1% (2/18).

## Discussion

Currently, no medications are indicated for the treatment of HPV infections that do not manifest as advanced precancerous lesions. The standard therapeutic approach for infected individuals is observation until spontaneous remission or progression to precancerous lesions (the "wait-and-see" approach). If the infection progresses and cervical lesions evolve to HSIL/CIN2 or CIN3, excisional or destructive treatments are required, which often involve invasive surgical procedures [10]. It has long been established that a deficiency in dietary proteins or amino acids can impair immunological function and increase susceptibility to infectious diseases [23]. Recent studies have highlighted the crucial role of amino acids in immune responses when regulating different processes. Amino acids have been shown to be essential to activate cytotoxic T and B lymphocytes, NK cells, and macrophages. Furthermore, they are involved in regulating the cellular redox state and the gene expression and proliferation of lymphocytes. Amino acids are also necessary for the production of antibodies, cytokines, and other cytotoxic substances [24].

The supplementation treatment used in this study is based on casein hydrolysate formula, a source of 18 amino acids (10 essential and eight non-essential, including lysine and arginine) in the form of dipeptides and tripeptides, as well as micronutrients such as vitamin A, folate, and zinc. Notably, peptides derived from casein have been shown to exhibit an immunomodulatory action. Furthermore, multifunctional peptides encrypted within their amino acid sequence are activated after a specific hydrolysis [25,26]. Beyond the importance of macronutrients for immune function, an adequate supply of micronutrients, such as minerals and vitamins, is also essential. Specifically, the contribution of micronutrients such as vitamin A, folates, and zinc has been directly associated with the immune response to HPV infections [16-18,27,28].

Vitamin A and folate have been associated with a protective effect against the development of cervical neoplasia in the context of persistent HPV infection [16]. Deficiencies of carotenoids such as vitamin A are clearly linked with the onset of cervical cancer and its precursor lesions, and folate deficiencies are associated with the occurrence of precancerous lesions of the cervix [18]. In a study involving 1248 men with HPV infection, it was observed that patients who consumed more retinol, vitamin A, and folates had a lower persistence of HPV infections [17]. Folates could play a role in regulating the integration and stability of the viral genome, thanks to their involvement in DNA synthesis, repair, and methylation [16,27]. Furthermore, both folate and vitamin A can inhibit cell proliferation, prevent DNA damage, and enhance immunological functions [16,27,28].

Zinc is an essential mineral that plays a crucial role in the proper functioning of the immune system. It regulates the intracellular signaling pathways of the cells of the innate and adaptive immune system. Additionally, it exhibits anti-inflammatory and antioxidant properties [29]. Regarding its importance in HPV management, zinc deficiency has been linked to increased persistence, progression, and recurrence of warts in patients with HPV [20]. Clinical studies have demonstrated efficacy using both oral and topical zinc in the treatment of warts [19,21].

In this study, supplementation with the casein hydrolysate-based formula demonstrated high rates of HR-HPV clearance at six months, regardless of having cervical lesions or not. Comparing supplemented participants to the control group, a significant difference was observed between the rates of total or partial HR-HPV clearance at six months (74.6% vs. 35.6%, respectively). Our results are in line with the findings from Bulkmans and colleagues who evaluated 44102 women. They reported spontaneous HR-HPV clearance rates of only 29% at six months and 41% at 18 months [30]. The control group in this study exhibited HR-HPV clearance rates like those observed in the general population, while participants receiving supplementation demonstrated nearly double the clearance rates across different groups.

It is well-established that more than 30% of patients with cervical HPV may present with multiple simultaneous infections [31-33]. Therefore, achieving total HR-HPV clearance, meaning that a patient negativizes the test for all HR-HPV types detected at baseline, is of great clinical importance. In this study, a significantly higher rate of total HR-HPV clearance was observed at six months in the supplemented group compared to the control group (50.7% vs. 26.7%, respectively, p<0.05). This suggests that supplementation with the casein hydrolysate-based formula is associated with a statistically significant ability to clear infections with multiple HPV types. Over half of HR-HPV-positive women have experienced a complete response after supplementation treatment.

When examining the evolution of intracervical lesions associated with HR-HPV infection at six months, no lesions were observed in participants who had normal cytology at baseline (HR(+)/Cyto(-)) after supplementation treatment, compared with those without treatment (28.6%). Notably, in participants with altered cytology (HR(+)/Cyto(+)) at baseline, lesion remission was achieved by a significantly greater number of participants receiving supplementation treatment compared with those without treatment (67.4% vs. 41.9%, respectively, p<0.05). A lower trend of progression toward HSIL lesions was also observed in the supplementation treatment group compared to the control group (4.7% vs. 9.7%, respectively). Thus, beyond preventing the appearance of HPV-associated cytological lesions and their progression, supplementation treatment with the casein hydrolysate-based formula in HR-HPV-positive women appears to be beneficial in improving the remission rate of existing low-grade lesions. 

It should be noted that observational studies conducted in a general HR-HPV-positive population without any kind of intervention have shown LSIL lesion persistence in up to 40% of cases after two years [34]. Several factors, including HPV type, multiple HPV infections, viral load, women’s age, and lifestyle, are associated with an increased risk of LSIL persistence [6,34,35]. Importantly, the persistence of HR-HPV is associated with a higher risk of lesion progression to precancerous lesions and cervical cancer [35]. Therefore, the ability to reduce HR-HPV persistence or the persistence of derived lesions through food supplementation may help reduce the risk of cervical cancer in HR-HPV-infected patients, especially when no alternative management options exist beyond the standard clinical wait-and-see approach. The encouraging results observed in this study, after only six months of casein hydrolysate-based formula supplementation, suggest its potential as a beneficial intervention.

Interestingly, approximately half of the participants with low-grade lesions at the beginning of the study who achieved lesion remission were also able to achieve total clearance of HR-HPV types at six months (67.4% of lesion remission and 39.6% of total HR-HPV clearance in the supplementation treatment group, and 41.9% and 22.6% in the control group, respectively). This disparity likely arises from the fact that the resolution of the lesions depends mainly on the participant's response to the infection, whereas the detection of the viral DNA depends on both the persistence of the infection and the persistence of exposure to the virus. Consequently, controlling viral exposure through partner management could significantly enhance treatment outcomes. This highlights the need to evaluate the benefit of nutritional supplementation for both participants and their sexual partners. Further studies, in which sexual partners receive nutritional supplementation, will be necessary to evaluate this hypothesis.

The observed increase in cellular immune effectors, such as NK cells and CD4+ and CD8+ lymphocytes, in this study should be highlighted. We must consider that, unlike the humoral immune response against HPV, which prevents infection by the virus, the clearance of HPV infections is primarily mediated by the cell-mediated immune response [36,37]. In this sense, T-cell homing and T-cell infiltration at the site of infection are related to immune control and the clearance of HPV infections [38]. However, there are times when the immune system is unable to control the progression of HPV infections or prevent recurrences in patients who have already been treated. Notably, NK cell deficiency has been linked to an increased susceptibility to HPV infections, a loss of control over them, and a higher risk of cervical cancer [39-42]. Given this, it is logical to think that helping the systemic cell-mediated immune system fight HPV could improve the prognosis of infections caused by this virus. Despite data collection limitations, it is likely that the positive impact observed is attributable to the ability of the nutritional supplement to improve the participants' immune system control of HPV infection, accelerate the clearance of the virus, improve the remission rate of its derived lesions, and offer additional general health benefits. Future-specific clinical studies will be necessary to establish a causal relationship between the improved immune parameters and the results observed in these participants. This study had limitations, including its prospective nature and the lack of randomization. Furthermore, supplementation decisions were based on physicians' clinical criteria, which could differ depending on the dedication of the different sites. Additionally, the lack of comprehensive demographic and anamnestic data collection in the studied population groups precludes a thorough assessment of treatment efficacy before study entry.

## Conclusions

This prospective, multicenter, controlled observational study demonstrates that supplementation with a casein hydrolysate-based formula, HuPaVir®, is a safe, well-tolerated, and easily administered oral nutritional support strategy for HR-HPV-positive women. Daily supplementation resulted in significantly higher rates of virus clearance and cervical lesion remission after six months compared to the conventional 'wait-and-see' approach used in clinical practice for women with HR-HPV. However, a large multicenter, randomized, controlled study with a larger number of patients and a longer duration is necessary to further validate these findings and establish a clear cause-and-effect relationship.
